# CALL A TEENAGER . . . THAT’S WHAT I DO!: GRANDCHILDREN HELP OLDER ADULTS USE NEW TECHNOLOGIES

**DOI:** 10.1093/geroni/igz038.1205

**Published:** 2019-11-08

**Authors:** Jennifer D Portz, Christine Fruhauf, Sheana Bull, Rebecca S Boxer, David Bekelman, Alejandra Casillas, Kathy Gleason, Elizabeth Bayliss

**Affiliations:** 1 University of Colorado, Aurora, Colorado, United States; 2 Colorado State University, Fort Collins, Colorado, United States; 3 Kaiser Permanente Institute for Health Research, Aurora, Colorado, United States; 4 UCLA, LA, California, United States; 5 Kaiser Permanente, Aurora, Colorado, United States; 6 Kaiser Permanente Colorado, Aurora, Colorado, United States

## Abstract

As older adults increasingly show interest in technology for their well-being, families will play an important role in promoting the adoption and use of beneficial health technologies. The purpose of this study was to conduct a sub-analysis of data collected from a large-scale qualitative project regarding older adults’ experiences using health information technology. Specifically, the sub-analysis explored older adults’ experiences with technology support from family members to inform strategies for promoting older adult engagement with new health technologies. While the primary analysis of the original study was theoretically driven, this paper reports results from the inductive, open-coding analysis. Twenty-four older patients (≥65 years) with multiple chronic conditions (Charlson Comorbidity Index > 2) participated in a focus group conducted at patients’ primary clinic. While conducting the primary theoretically-driven analysis, coders also utilized an open-coding approach to ensure important ideas not reflected in the theoretical code-book were captured. Open-coding resulted in a primary theme, “family support”, that was furthered categorized by who and how the tech-support was provided. Participants were not specifically asked about family support, yet family assistance and encouragement for technology emerged from every focus group. Participants repeatedly mentioned that they called their grandchildren and adult children for help with technology. Participants also reported that family members experienced difficulty when teaching technology use. Family members struggled to explain simple technology tasks and were frustrated by the slow teaching process. Family support, specifically via grandchildren, may have a key role in the successful adoption and use of emerging health technologies.

